# Archaeological and Chemical Investigation on Mortars and Bricks from a Necropolis in Braga, Northwest of Portugal

**DOI:** 10.3390/ma14216290

**Published:** 2021-10-22

**Authors:** Ana Fragata, Carla Candeias, Jorge Ribeiro, Cristina Braga, Luís Fontes, Ana Velosa, Fernando Rocha

**Affiliations:** 1 GeoBioTec—GeoBioSciences, GeoTechnologies and GeoEngineering Research Unit, Geosciences Department, University of Aveiro, 3810-193 Aveiro, Portugal; candeias@ua.pt (C.C.); tavares.rocha@ua.pt (F.R.); 2 Lab2PT—Landscapes, Heritage and Territory Laboratory Research Unit, Institute of Social Sciences, University of Minho, 4710-057 Braga, Portugal; jribeiro@uaum.uminho.pt (J.R.); cristinabraga@era-arqueologia.pt (C.B.); luisfontes1959@gmail.com (L.F.); 3 RISCO—Research Centre for Risks and Sustainability in Construction, Department of Civil Engineering, University of Aveiro, 3810-193 Aveiro, Portugal; avelosa@ua.pt

**Keywords:** mortars, roman bricks, *Bracara Augusta*, necropolis, funerary nucleus, archaeological sites, chemical characterization

## Abstract

This investigation intends to study and characterize the mortars and bricks from walls and floors used in the funerary nucleus of the archaeological site of Dr. Gonçalo Sampaio Street (Braga, Portugal), associated with the Via XVII necropolis of the *Bracara Augusta* Roman city. The diversity of the funeral structures and their exceptional state of conservation make this sector of the necropolis an unprecedented case and a reference site in the archaeology of Braga, a determinant for its conservation and musealization. Nineteen mortars samples were analysed by X-ray Fluorescence. The results showed clear chemical composition differences among coating and floor mortars (CFM), masonry mortars (MM) and bricks (B) groups of samples. The chemical affinity between CFM from the V to IV centuries, CFM from the IV to V centuries, MM from brick walls (IV–V centuries), MM from stone walls (V–VII centuries) and B from the IV to V centuries samples were confirmed by statistical analyses. Their composition was distinctly related to the use of different raw materials, according to their chronological context; in mortars, according to their function in the structures; and in some samples, from contamination.

## 1. Introduction

Mortars are an anthropic material made of binder and aggregates, with a fundamental role in the construction of ancient buildings. The study of Roman mortars and bricks from archaeological sites provides important information on the composition and execution techniques of those highly durable materials. In Portugal, Roman mortars from Beja-Pisões, Braga, Conimbriga, Marvão-Ammaia and Tróia [1,2,3,4,5] and bricks and clayed ceramic materials (CCM) [6,7,8] were investigated, regarding the preservation, conservation, and archaeological perspectives. Specifically, there are some investigations on necropolis archaeological sites from Jordan [9], France [10,11] and Portugal [12] from an archaeological perspective, and in Italy [13,14] and Spain [15] from a materials characterization perspective.

The archaeological funerary site of Dr. Gonçalo Sampaio Street, excavated by the Archaeology Unit of the University of Minho (Braga, Portugal), is an excellent case study of preserved mortars in masonry constructions (stone and brick), but also in different levels of *opus signinum*, applied in the diverse documented graves. Additionally, it allowed the analysis of the lateritic materials used in the structures.

## 2. Archaeological Setting

**Figure 1:**
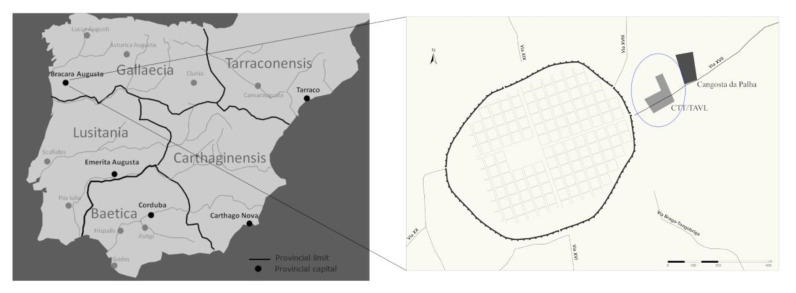
Location of the studied area of Via XVII necropolis in the Roman urban layout, on the Iberian Peninsula. Adapted from [6,17].

*Bracara Augusta*, the city. The story of *Bracara Augusta* began in Roman times. The city was founded by Emperor *Augustus* around 16–15 BC in the NW of *Hispania* [16] (Figure 1). Convent capital of the *Tarraconensis* province, the city became the capital of the new province of the *Gallaecia* under Diocletian. During the V and VI centuries, waves of Germanic tribes swept into the former Roman territories, amongst them the Suevi and the Visigoths. The Suevic Kingdom adopted Braga as its capital in 411 AD, having been absorbed by the Visigothic Kingdom in 585 AD [17]. The political changes and the disturbances generated did not prevent the city from maintaining a remarkable economic and constructive dynamism.

*Bracara Augusta*, the necropolis. The city had several necropolis spaces that, as usual in Roman cities, were located outside the walls, associated with main and secondary roads. Thus, in Braga, the Maximinus necropolis (Via XX and XVI), the Rodovia necropolis (*Via Bracara Augusta*—*Emerita Augusta*), the Campo da Vinha nuclei (Via XIX/ XVIII?) and the necropolis of Via XVII (Via *Bracara Augusta*—*Asturica Augusta*), revealed a recent building intervention, forming the archaeological nucleus of Dr. Gonçalo Sampaio Street and the nucleus of Cangosta da Palha [18] and references therein (Figure 1).

The study of the Roman necropolises of Braga resulted from the archaeological record accumulated over nearly 40 years of research in the city, in the context of the *Bracara Augusta* project [16], under the responsibility of the Archaeology Unit of the University of Minho. Several studies resulted from this research, among which one dedicated to the theme of the necropolis nucleus of Via XVII [19], and another one related to the funerary topography of the Via XVII necropolis in Late Antiquity [18]. The Via XVII necropolis, located in one of the East exits of *Bracara Augusta*, known since the 1940s, is undoubtedly the most studied ancient funerary context in the city, of which 12 distinct nuclei are known, dating from the Roman period to the Suevo–Visigothic era.

*A nucleus of the* Via *XVII Necropolis.* Between the end of 2007 and May 2009, a building from the beginning of the 20th century, and its surrounding area, with a total area of 5600 m^2^, was subjected to a major urban rehabilitation project, in the context of which a major archaeological intervention took place. The need to build an underground car park implied the excavation of the area and the archaeological work for over two years. The excavation work of this nucleus was completed in November 2016.

The work carried out led to the identification of a large necropolis area, a section of the Via XVII and a glass workshop, which was active between the Lower Roman Empire and the Suevo–Visigothic period. Regarding the identified necropolis area (integrated into the Via XVII necropolis), it is a large burial space with 129 incineration graves, 94 *ustrinae*, 65 burials, 4 mausoleums and 6 funerary enclosures, with a wide chronology of use, between the first century and the Late Antiquity (V–VII centuries) [17,20].

**Figure 2:**
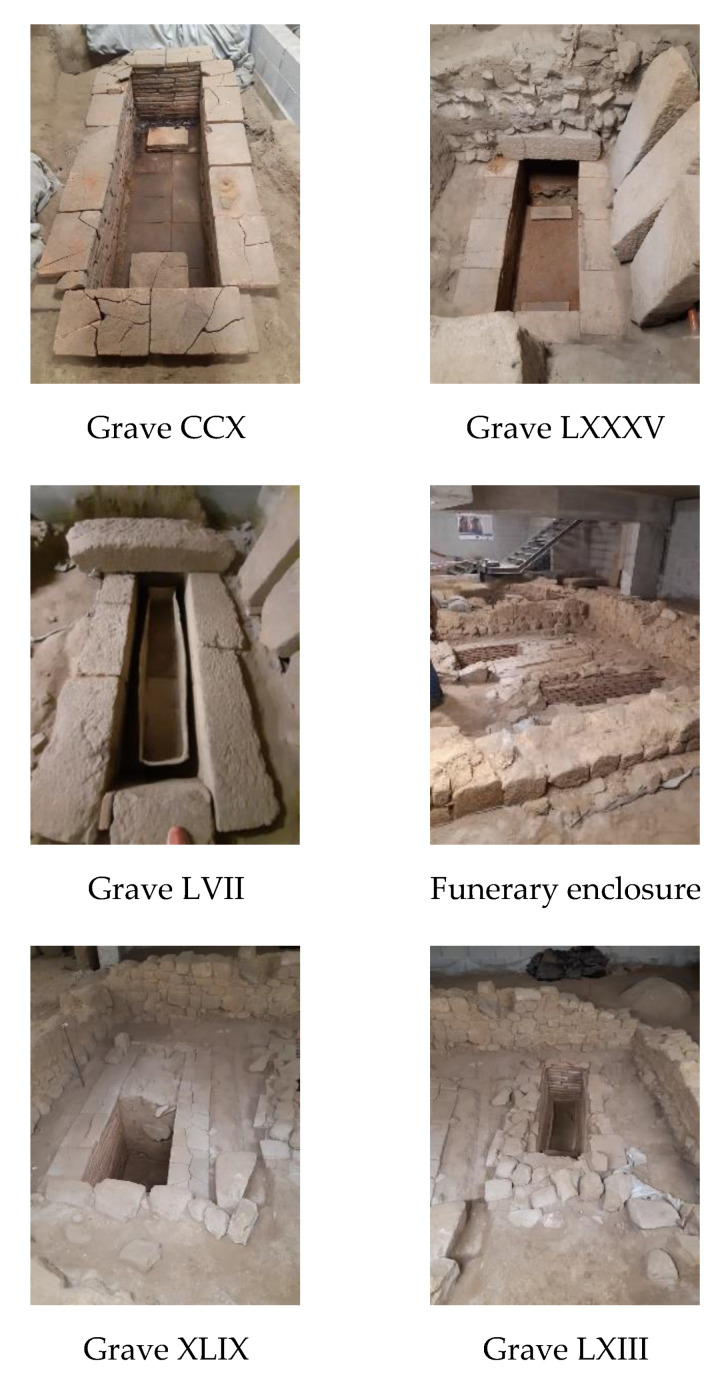

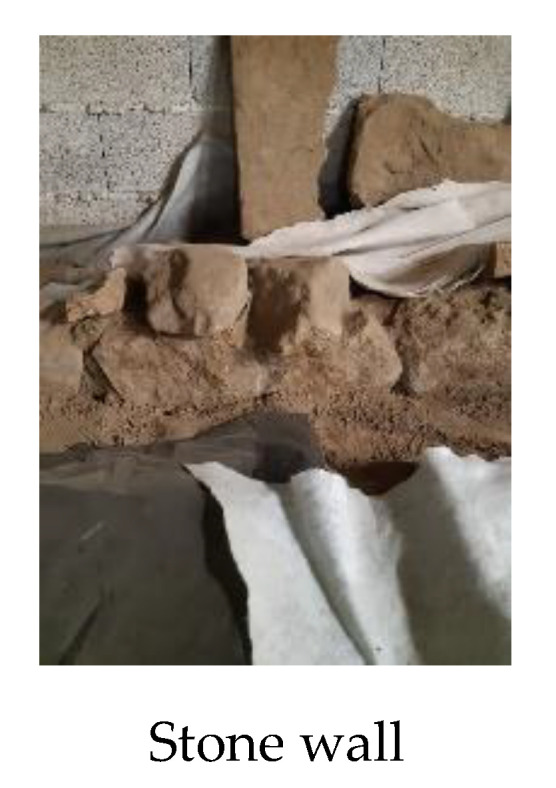
Funerary enclosure, graves and stone wall of the funerary nucleus.

Nowadays, the only nucleus of a necropolis accessible in Braga occupies an area of reduced dimensions, in which five burial graves were discovered, identified (from west to east, and from north to south) as LXIII, XLIX, CCX, LXXXV and LVII, presenting differentiated constructive orientations and techniques, dated from the IV to VII centuries. There is also a rectangular enclosure built with masonry walls, which partially overlaps the graves XLIX and LXIII, and a stone masonry wall oriented SE–NO, which seemed to delimit the area to the west [20] (Figure 2). The funerary enclosure was assumed to be constructed between the V and VII centuries, later than the graves XLIX and LXIII, from the IV to V centuries. The chronology was attributed through the relative dating methodology, based on the analysis of the materials collected in the intervention, namely ceramics, glasses and metals, considering their evolution according to the stratigraphic levels [21].

### Description of the Structures Identified

**Figure 3:**
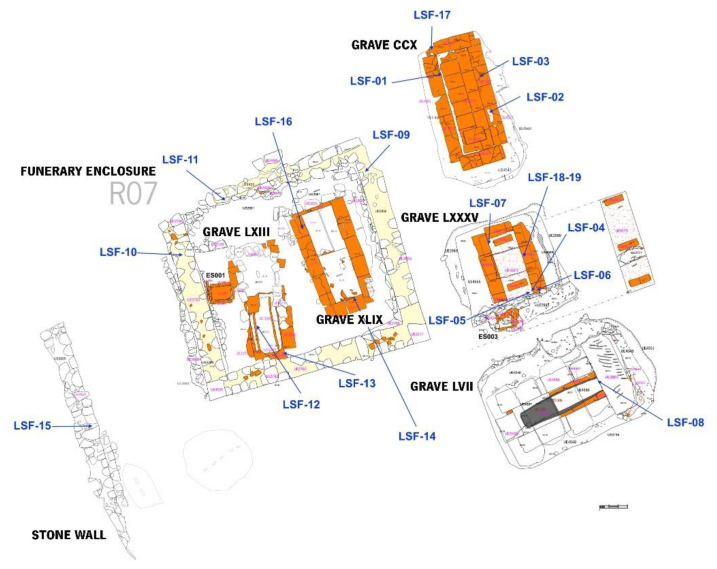
Schematic representation of the funerary nucleus and sampling areas. Adapted from [21].

A schematic representation of the graves LXIII, XLIX, CCX, LXXXV and LVII, the funerary enclosure and the stone wall can be found in Figure 3.

Grave LXIII is formed by a rectangular box, oriented N–S, with an implantation area of 3.94 m^2^ and 2.08 × 0.54 × 0.54 m. It is entirely made of bricks, mostly of the *lydion* type (0.45 × 0.30 × 0.05 m), and it sits directly on the granitic alterite, adapted for this purpose. The size, configuration and the absence of spoils of the grave point to it being a burial grave dating from the IV to V centuries.

Grave XLIX is located to the east of LXIII grave, and has a roughly rectangular box, oriented N/NW–S/SE, with an implantation area of 5.4 m^2^ and internal measures of 2.08 × 0.60 − 0.67 × 0.9 m. Its construction is also made of brick masonry, of the *lydion* type, being delimited on the surface by a back row of granitic elements. The base consists of large well-cut granite slabs, covered with a reddish mortar, such as *opus signinum*, penetrating the interstices of the stones. The proposed chronology is of the same period as the LXIII grave.

Grave CCX is located NE of the above graves, with an NNO–SSE orientation. It was discovered in 2009 but only fully excavated in 2016. It is a box with walls and a base made of brick, mostly of the *lydion* typology, covered with a mortar-like *opus signinum*. On the bottom, at each end of the box, *lydion* bricks establish a platform for laying the stretcher in such a way that allows the collection of the moorings. The cover is made of six granitic stones with different dimensions, with the flat face facing inwards. Its proposed chronology is the same as from LXIII and XLIX graves.

Grave LXXXV was located at the south of CCX grave with an NNO–SSE orientation. It was discovered in 2009 but only fully excavated in 2016. It had an implantation area of 6.9 m^2^ and an interior span of 2.10 × 0.59 × 0.54 m. This grave is in a brick box, with the interior coated with an *opus signinum*-type mortar, closed with granitic elements. On the structure’s leveling embankment, a brick box was documented. It was closed with large granitic blocks, with the joints sealed with a mortar-like *opus signinum*, occasionally linked to the burial signal. It was dated from the V–VII centuries.

Grave LVII is located to the south of the LXXXV grave. It is a box construction with a rectangular plan, oriented OSO–ENE, made from granitic masonry, well squared and with smooth flanks. It has an area of deployment of 8.8 m^2^ and an interior span of 2.5 × 0.60 × 0.60 m. The cover is made of monolithic granitic slabs, sealed with a mortar-like *opus signinum*. Inside the grave, a rare rectangular container made of lead (Pb) was found, which was considered a unique archaeological feature in the Iberian Peninsula, despite other examples known [10,11]. In the excavation, the occurrence of water infiltration and possibly the contamination of the grave materials was observed. The presence of fine materials was also observed. As for its chronology, it was suggested between the V and VII centuries.

The west wall of the enclosure separated the necropolis (II century), located to the west of the funerary nucleus, showing some deterioration and other nuclei with different characteristics.

Lastly, the funerary enclosure is a quadrangular building, quite flat, made of granitic stone, situated about 28 m north of the Roman road Via XVII with an NNO–SSE orientation and occupying an area of 15.12 m^2^, with 3.98 × 3.80 m. The perimeter walls are preserved, which rest on footings made of small stones arranged in an irregular shape. It is made of asymmetrical masonry, showing two-row blocks with irregular size and shape. At the SE and SW limits, the walls sit on parallelepiped granitic ashlars. The laying mortar, as well as that of the joints, has clay characteristics and a yellowish color. The enclosure contains two previous graves (LXIII and XLIX), possibly functioning as an appropriation of important funerary space. It was dated between the V and VII centuries.

The value of this set, with its diversity of burials and rarity, particularly regarding the lead coffin identified in the grave LVII, led to its *in situ* conservation and future musealization.

The graves’ material samples were taken from the different types of constructions identified according to a multidisciplinary approach, archaeological and geochemical, by the evidence of typo chronologies to determine whether certain characteristics are related to their use, the existing binders, to characterize geochemically the materials and to compare them with other cases studied.

The aim of this study was to characterize the mortars and bricks to find differences and/or similarities considering the type of mortars/function in the structures and the period of construction. Two types of mortars were considered: the coating and floor mortars, with a reddish color that can be associated with ceramic inclusions, and the masonry mortars with a light brown tone, in general, with some samples being brown but all without ceramic inclusions.

## 3. Materials and Methods

### 3.1. Samples Collection

For this study, 19 mortar and brick samples were collected from different structures of the funerary nucleus, graves, funerary enclosure and the dividing wall. Sampling was undertaken considering the structure’s function, the macroscopic characteristics, and the available material without destroying the funerary site (Table 1 and Figure 3). The coating and floor mortars (CFM) from the graves, between the IV–V and V–VII centuries, were removed from the internal renders of brick walls (samples SF01 and SF07), the stone wall (SF08), the top of the covers (SF02 and SF06), the uppermost layer (SF04) and the lowermost layer (SF05). These last two were from the grave floor. The masonry mortars (MM) were taken from bedding mortars of graves’ brick walls (SF03, SF12, SF16 and SF19) and stone walls (SF09, SF10, SF11 and SF15), attributed to different chronologies, II, IV–V and V–VII centuries. Brick samples from the walls showed different chronological contexts between the IV–V (SF13, SF14 and SF17) and the V–VIII (SF18) centuries.

### 3.2. Chemical Analysis by X-ray Fluorescence (XRF)

The present study focuses on the chemical composition of the collected mortars and bricks. The chemical analyses were performed on crushed samples by X-ray fluorescence (XRF) carried out by a Panalytical Axios spectrometer PW4400/40 X-ray (Marvel Panalytical, Almelo, The Netherlands) operating on an Rh tube under argon/methane at the GeoBioTec/University of Aveiro laboratories. For major and minor elemental analysis, the Omnian 37 and Pro-Trace2021 software were used, respectively. Loss on Ignition (LOI) was determined by heating the samples at 1000 °C with an electric furnace for 3 h. The major elements analysed with a detection limit of 1%, were: aluminum oxide (Al_2_O_3_), calcium oxide (CaO), iron oxide (Fe_2_O_3_), potassium oxide (K_2_O), magnesium oxide (MgO), manganese oxide (MnO), sodium oxide (Na_2_O), phosporus oxide (P_2_O_5_), silicon oxide (SiO_2_), sulfur oxide (SO_3_) and titanium oxide (TiO_2_). For the trace elements analysed, the detection limits were: arsenic (As) = 4.06 mg/kg, barium (Ba) = 6.90 mg/kg, bromine (Br) = 0.78 mg/kg, chloride (Cl) = 20 mg/kg, chromium (Cr) = 1.96 mg/kg, copper (Cu) = 2.84 mg/kg, niobium (Nb) = 0.84 mg/kg, neodymium (Nd) = 2.00 mg/kg, nickel (Ni) = 2.00 mg/kg, lead (Pb) = 1.72 mg/kg, tin (Sn) = 3.02 mg/kg, strontium (Sr) = 0.72 mg/kg, vanadium (V) = 2.78 mg/kg, yttrium (Y) = 0.86 mg/kg, zinc (Zn) = 1.28 mg/kg and zirconium (Zr) = 0.80 mg/kg. The precision and the accuracy of analyses and procedures were monitored using internal standards, adopting quality control blanks and certified reference material. The confidence limits of the results were 95% and the relative standard deviation was between 5% and 10%.

### 3.3. Chemical Statistical Analysis

Variables were processed using IBM SPSS^®^ statistics v25. The normality of the data was verified by the Shapiro–Wilk test (*p* > 0.05). In order to confirm the groups and determine the statistically significant differences (*p* < 0.05), ANOVA, cluster, discriminant analyses, Tukey’s test, Student’s *t*-test and k-means were used.

## 4. Results and Discussion

The mortar and brick samples’ chemical composition is detailed in Table 2 and Table 3. Coating and floor mortars group (CFM) include all *opus signinum* mortars and SF05 sample from the preparatory layer of LXXXV grave’s floor, showing higher mean values of Al_2_O_3_, Fe_2_O_3_, P_2_O_5_, SO_3_, As, Br, Cl, Cr, Nb, Ni, Sn, Y and Zn than masonry mortars (MM). Bricks (B) were characterized with higher content on Al_2_O_3_ and Fe_2_O_3_ and K_2_O, As, Nb, Ni, Sn, Y and Zr and lower content on CaO, MgO, MnO, Na_2_O, P_2_O_5_, SO_3_, TiO_2_, Ba, Cl, Cu, Nd, Pb, Sr and V when compared to CFM and MM samples. The mortars and bricks’ low CaO content [1,22] support the idea of the use of locally available raw materials, as the Braga region is dominated by granites [23]. Additionally, in mortars, the high SiO_2_ concentration is directly related to the low CaO and MgO contents, confirming the absence of calcareous/dolomitic aggregates (Table 2). The Fe_2_O_3_ mortars content did not reveal a significant variation (5.37–7.61%, except for SF02 with the lowest content among mortars—3.51%). Among mortars, the higher Fe_2_O_3_ content (between 5.54% and 7.61%) in *opus signinum* ones (cocciopesto mortars) from CFM (except SF01 and SF02, with lower content) can be related to the presence of red ceramic fragments or powder, which may result, although in lower quantity, from the aggregate. The higher aluminate content in those mortars confirms this idea, as well as the most abundant presence of ceramic fragments in SF04, SF06, SF07 and SF08). However, MM displayed relatively high contents of Fe_2_O_3_ suggesting that they came from the aggregate in this case.

Brick samples, ranging from 6.33 to 8.52% of Fe_2_O_3_, also did not reveal significant variation (*p* > 0.05); although, a relatively higher content could have been the result of Fe_2_O_3_ application to facilitate bricks’ firing by increasing the heating storage capacity. The lowest LOI content was observed in bricks SF14 and SF17, with 1.62 and 1.36%, respectively, which can be related to their high firing temperature and higher kaolinitic content. The chemical composition of the raw clay used in bricks is relatively uniform, as the variability means of Al_2_O_3_ (30.17%) and SiO_2_ (52.07%) exhibit low variance, with a standard deviation of 2.01–2.65%, respectively (Table 2). Low content of CaO, Na_2_O and TiO_2_ was found in all brick samples, although the first two can be associated with contamination by lime mortars and soluble salts [24].

The high content of Cu and Pb in mortars can possibly be the result of the considerable degree of exposure to the modern construction materials (concrete and Portland cement) (e.g., [25]) that were used above this archaeological site. Considerably higher contents of Cu and Pb were observed in mortar samples SF01 (Pb = 180 mg/kg), SF03 (Cu = 210 mg/kg), SF09 (Cu = 150 mg/kg, Pb = 190 mg/kg) and SF15 (Cu = 87.5 mg/kg, Pb = 220 mg/kg), which are closer to those modern materials (Table 3), as result of contamination. A remarkable Pb content of 17,890 mg/kg was found in sample SF08, a coating mortar from grave LVII, that might be related to contamination due to a leaden coffin found in the grave with signs of corrosion related to water infiltration.

The Cl and Ba contents, although low, found in bricks (Table 3) may result from contamination. As with clayed bricks, those elements are not expected due to the firing process. The presence of Cl in those samples (except in SF14, in which Cl content was below the detection limit) can be ascribed to infiltration and/or capillary rise that may transport these soluble salts through those porous materials [26].

**Figure 4:**
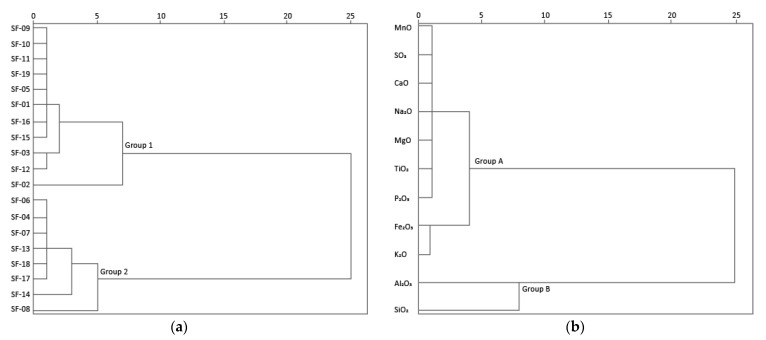
Cluster analysis of mortars and bricks samples major element concentrations: (**a**) samples, and (**b**) variables.

The sample’s chemical content was explored using multivariate cluster analyses. The samples major elements dendrogram revealed two major clusters (Figure 4a): Group 1 samples, which included all masonry mortars (MM) and three coating and floor mortars (CFM) samples (SF01, SF05 and SF02), a set enriched in SiO_2_ (50.07–63.83%), MgO (1.43–1.58%) and K_2_O (5.80–6.75%). The higher MgO content in group 1 (1.43–1.58%, except sample SF02), when compared to group 2, can be related to the need to increase plasticity through the addition of more plastic clays (poorer in kaolinite, richer on illite/smectite); however, it can also be ascribed to lower quality (less kaolinitic) of raw materials used, or to mortars degradation. The outlier SF02 sample, removed from a structure previously consolidated with paraloid, showed the highest SiO_2_ (63.83%) and the lowest K_2_O (5.45%) and Al_2_O_3_ (18.28%) content among group 1. Group 2 includes all brick (B) samples and the remaining CFM samples (SF04, SF06, SF07 and SF08—all *opus signinum* from the V to VII centuries), a set of samples enriched in Al_2_O_3_. The higher Al_2_O_3_ (28.27–32.83%) content can be ascribed to Al-rich kaolinitic clays, which highlighted the careful selection of locally (Braga region is very rich in kaolin deposits) raw materials on those materials. SF08 was revealed to be an outlier in group 2, with the lowest SiO_2_ and K_2_O contents among the group samples, which may be the result of previous contamination with fine materials. By considering the variable cluster analysis of all the samples, Al_2_O_3_ and SiO_2_ concentrations were distinguished from the other major elements analysed, forming a distinctive cluster (Figure 4b). A subgroup of Group A included Fe_2_O_3_ and K_2_O, with bricks showing lower K_2_O and higher Fe_2_O_3_ than mortars.

Cluster analysis of CFM, MM and B groups’ major elements content was also performed individually. On CFM samples, two clusters were identified, one cluster composed by SF08 with an affinity to SF04, SF06, and SF08 (V–VII centuries), with higher Fe_2_O_3_ and Al_2_O_3_ (that can be ascribed to the higher inclusion of brick fragments) than the other cluster with SF05 (V–VII centuries), and SF01 and SF02 (IV–V centuries). The cluster analyses of MM group, samples SF03 and SF12, revealed a distinct composition, with lower SiO_2_ and Al_2_O_3_ content, from SF15. In the B group, sample SF14 presented a slight distinct composition from the remaining brick samples, with higher SiO_2_, MgO and TiO_2_ and lower P_2_O_5_ content.

**Figure 5:**
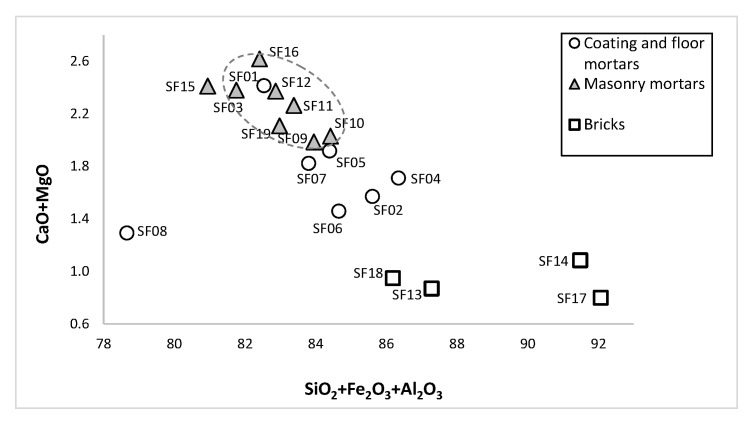
Mortars and bricks CaO + MgO vs. SiO_2_ + Fe_2_O_3_ + Al_2_O_3_ binary diagram.

The diagram CaO + MgO vs. SiO_2_ + Fe_2_O_3_ + Al_2_O_3_ can be roughly related to the presence of binder and aggregate [27], respectively, since these mortars do not present calcareous aggregates. Mortars formed two groups (Figure 5): a group composed by all MM and one CFM (SF01) with a good compositional homogeneity, in the upper part of the diagram, with less binder (2.0–2.6%) and higher aggregate (80.9–84.4%) content; another group was composed by the remaining CFM samples, despite SF05 and SF07, with binder content similar to MM samples (1.9 and 1.8%, respectively). Sample SF08 revealed the lowest binder and aggregate content among mortars, possibly due to contamination with fine materials. Mortars displayed higher CaO (0.35–0.99%) and MgO (0.90–1.66%) content than bricks CaO (0.12–0.33%) and MgO (0.11–0.54%). The higher MgO content in MM samples may be the result of the application of Mg-enriched clays (non-pure kaolin) to increase mortars’ plasticity (pure kaolins are low plastic clays). Additionally, in CFM samples (*opus signinum* mortars with ceramic powder or fragments), an increased content of SiO_2_ + Al_2_O_3_ + Fe_2_O_3_ was observed when compared to masonry mortars, suggesting the use of pozzolanic materials and, therefore, the hydraulic characteristics of these mortars. Although a good degree of hydraulicity of the mortar could be attained using finely grounded bricks, if produced with poor clay raw materials, as was this case, the pozzolanic reaction could not occur due to the low amounts of amorphous materials [22,28]. Bricks showed lower binder content (0.8–1.1%) than mortars, and their aggregate content clearly falls into two distinct groups: one more similar to CFM mortars (SF18 and SF13) and another with the highest aggregate content (SF14 and SF17). The binder and aggregate proportion allowed to distinguish CFM (*opus signinum* mortars) from the V to VII centuries, MM from stone walls (inside funerary enclosure) from the V to VII centuries, MM and CFM (both from brick walls) from the IV to V centuries, and bricks (except *lydion* brick).

**Figure 6:**
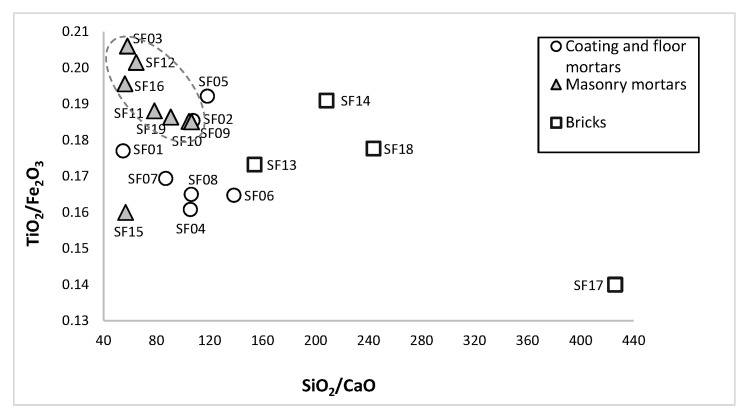
Mortars and bricks TiO_2_/Fe_2_O_3_ vs. SiO_2_/CaO binary diagram.

The binary diagram TiO_2_/Fe_2_O_3_ vs. SiO_2_/CaO showed a homogeneous group composed of MM samples, with low SiO_2_/CaO ratio, differing from CFM samples with lower TiO_2_/Fe_2_O_3_ ratio and bricks with higher SiO_2_/CaO ratio (Figure 6). The similar TiO_2_/Fe_2_O_3_ ratio (0.16–0.17%) of the CFM (SF04, SF07, SF08 and SF06) samples from the V to VII century construction phase suggested the use of the same raw materials. Similarly, MM samples from V to VII (SF09, SF10, SF11 and SF19) and from the IV to V centuries (SF03, SF12 and SF16) seemed to use different raw materials, according to the construction phase of the structures to which they belong. Sample SF15, a unique MM sample from the II century stone wall, was clearly separated from MM samples with affinity to CFM samples due to its higher content in Fe_2_O_3_ (7.13%), which may arise from the aggregate. The MM showed a more homogeneous chemical composition than CFM, and their lower content in Fe_2_O_3_ and higher content in TiO_2_ and MgO can be attributed to the raw materials used (binder and aggregates): a less pure lime may have been used, with higher content in magnesium combined with the fact that local materials with a particular composition may have been used. The high content of Fe_2_O_3_ in CFM (Table 2) can be attributed to the presence of ceramic particles, which may also come, although in smaller quantities, from the aggregate. Among CFM samples, the results pointed out to lower percentage of ceramic particles in samples SF01 and SF05 (Figure 5). Observing the percentage of aluminates in CFM samples, this idea was reinforced, as well as the more abundant presence of ceramic particles in samples SF04, SF06, SF07 and SF08, forming a distinct group (Figure 5). Bricks were more dispersed than mortars and showed the highest SiO_2_/CaO ratio (160–430%). The *lydion* brick sample (SF-17) differed in composition from other brick samples, showing the lowest TiO_2_/Fe_2_O_3_ ratio and CaO content (0.12%) and sample SF13 with the lowest SiO_2_/CaO content. Considering the relation in the binary diagram TiO_2_/Fe_2_O_3_ vs. SiO_2_/CaO, associated with the raw materials used, compositional differences were observed between CFM (*opus signinum* mortars from brick walls) from the V to VII centuries, MM (from stone walls inside the funerary enclosure) from the V to VII centuries, and MM (from brick walls) from the IV to V centuries.

**Figure 7:**
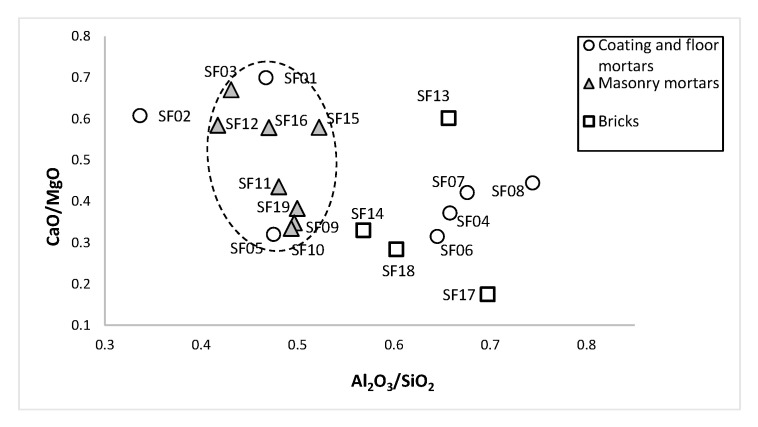
Mortars and bricks CaO/MgO vs. Al_2_O_3_/SiO_2_ binary diagram.

The CaO/MgO ratio showed some variability (Figure 7), which in some cases was not directly linked to the variation in calcium oxide, suggesting the use of binders from different sources. As a result, the binder materials used in MM samples SF09, SF10, SF11 and SF19 (V–VII centuries) and for CFM samples SF04, SF05, SF06, SF07 and SF08 (V–VII centuries) could have been the source. The CFM samples (SF01 and SF02) and MM samples (SF03, SF12, SF16) from the IV to V centuries showed a similar CaO/MgO ratio (between 0.58 and 0.70), suggesting the same material source. The MM sample SF15, from the II century, could have used the same binder materials source and different from samples from the IV–V centuries. The Al_2_O_3_/SiO_2_ ratio clearly differentiates two groups: one mainly composed of MM samples with lower Al_2_O_3_ content, and a group mainly composed of CFM samples (SF04, SF06, SF07 and SF08), suggesting a more careful selection of raw materials than other samples, as well the presence of brick particles on mortars composition. Bricks showed less homogeneous composition than mortars, although some similarities in the raw materials used could be found between SF14 and SF18 and between SF13 and SF17 samples. From the binary diagram CaO/MgO vs. Al_2_O_3_/SiO_2_ analyses, chemical differences were observed between CFM (*opus signinum* from brick walls) from the V–VII centuries, MM (from stone walls inside the funerary enclosure) and MM (from brick wall) both from the V–VII centuries, CFM (coating mortars from brick walls) from IV–V centuries and MM from the brick wall.

**Figure 8:**
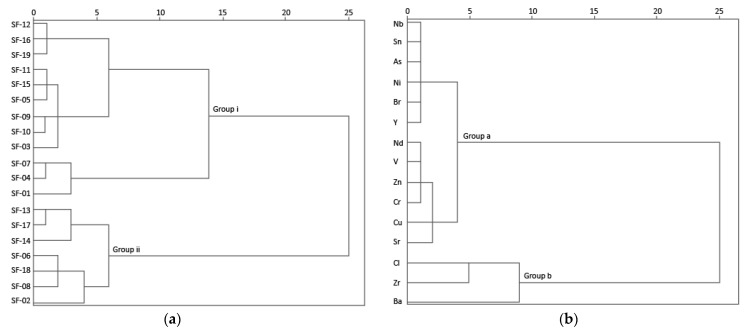
Cluster analysis of mortars and bricks samples trace element concentrations: (**a**) samples and (**b**) variables.

The cluster analysis of the trace elements composition showed two main samples associations (Figure 8a): group i, including MM samples and CFM SF01, SF04, SF05 and SF07 samples, and group ii, with bricks and the remaining CFM SF02, SF06 and SF08 samples. The variable cluster analysis revealed that Ba, Cl and Zr concentration trends were distinctive from the others, forming a distinctive cluster (Figure 8b). Excluding some samples, in general, group i showed higher Ba (500–900 mg/kg), Sr (86–180 mg/kg, except in SF04 and SF07 samples, with 69.2 and 77.6 mg/kg, respectively) and Cl (160–610 mg/kg, excluding SF03 and SF09 both with 120 mg/kg). In group ii, higher Zr (370–590 mg/kg, excluding SF02 with 180 mg/kg) content was found. The CFM samples SF01, SF04 and SF07 in group i (excluding SF05), formed a distinct subcluster, mainly due to its higher Cl and lower Ba and Sr contents. In group ii, the CFM samples SF02, SF06 and SF08 also formed a distinctive cluster (except for SF18, included in CFM subcluster) mainly due to their higher Ba (400–490 mg/kg) and Cl (160–280 mg/kg) and lower Zr (370–480 mg/kg, excluding SF02 with 180 mg/kg) content. Bricks showed the lowest Sr content (35.6–46.1 mg/kg excluding SF17 with 89.8 mg/kg) among all samples.

The individual trace elements cluster analysis of CFM, MM and bricks samples was performed. The CFM samples revealed two groups, a cluster with SF05 sample (V–VII centuries) revealing affinity to SF01 (IV–V centuries), SF04 and SF07 (V–VII centuries), with higher Ba (500–820 mg/kg) and Cl (470–610 mg/kg) contents than the other cluster formed with SF-08, SF-06 (V–VII centuries) and SF-02 (IV–V centuries) samples, with higher Zr (370–460 mg/kg, excluding SF02 with 180 mg/kg) content. In the analyses of the MM group, samples SF12, SF16 and SF19 (IV–V centuries) revealed a distinct composition from the other MM samples, showing higher Ba (800–900 mg/kg), Cl (320–370 mg/kg) and Sr (170–180 Mg/kg). The bricks group cluster analysis suggested that sample SF18 has a distinct composition from the remaining brick samples, with higher Cl (280 mg/kg) and V (67.6 mg/kg) content.

**Figure 9:**
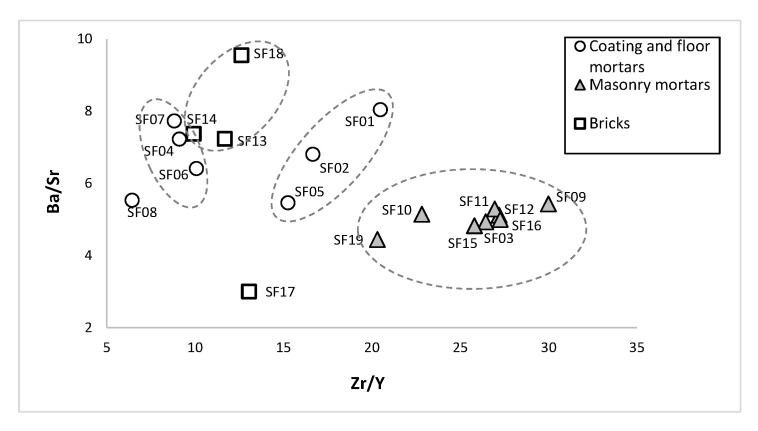
Mortars and bricks Ba/Sr vs. Zr/Y binary diagram.

The Ba/Sr vs. Zr/Y binary diagram (Figure 9) revealed information on the employed raw materials. Lower Ba/Sr and higher Zr/Y ratios suggest the presence of natural rock fragments rather than ceramic materials (cocciopesto) in the aggregate [29]. Bricks and CFM samples presented higher Ba/Sr and lower Zr/Y ratios, which can be attributed to the presence of ceramic compounds. The CFM samples SF04, SF06, SF07 and SF08 (V–VIII centuries) showed lower Zr/Y and higher Ba/Sr ratios, with higher Al_2_O_3_, Fe_2_O_3_ and TiO_2_ contents than the remaining CFM samples, which suggested their higher content in ceramic compounds. As expected, MM samples showed lower Ba/Sr and higher Zr/Y ratios. The SF13 and SF14 brick samples from the IV–V centuries showed similar Ba/Sr and Zr/Y ratios and suggested the use of the same source of raw materials. As a result, through the relation between Ba/Sr vs. Zr/Y, the compositional differences allowed to distinguish CFM (*opus signinum* from brick walls) from V–VII centuries and MM (from brick walls) from IV–V centuries.

## 5. Conclusions

The present study allowed us to confirm, in the Via XVII necropolis of the *Bracara Augusta* Roman city (Braga, Portugal), the distinctly chemical composition among coating and floor mortars (CFM), masonry mortars (MM) and bricks (B). The chemical affinity of each group (coating and floor mortars (CFM) from the V–VIII centuries, CFM from the IV–V centuries, masonry mortars (MM) from brick walls (IV–V centuries), MM from stone walls (V–VII centuries) and bricks ((B) from IV–V centuries) were confirmed by statistical analyses. Their composition was distinctly related to the use of different raw materials, according to their chronological context and, in mortars, according to their function in the structures and, in some samples, from contamination.

The mortar and brick low CaO content support the idea of using locally available poor-Ca raw materials. Additionally, a more careful selection of raw materials on bricks and CFM from V–VIII centuries was observed, using richer-Al kaolinitic clay, and the binder sources differed according to the different construction phases. A general higher compositional homogeneity was observed on MM compared to CFM. Some contamination was observed as a result of the proximity of some posterior funerary structures to the new building structure (made of Portland cement and concrete).

The characterization of the mortars and bricks from this funerary nucleus is the first step for the study of the provenance of the raw materials, the objective of future work. Moreover, the investigation on the compositional chemical data obtained for these original materials can be useful for the adequate reproduction of compatible mortars for conservation and restoration purposes, considering that this archaeological site is under musealization works.

## Figures and Tables

**Table 1:** Description of the mortars and bricks samples from the funerary nucleus.

	Sample	Location Description	Brick or Mortar Function in the Structure/Construction Technique	Construction Phase (Century)	Color/Consolidation/Conservation State
Masonry mortars (MM)	SF03	Grave CCX; brick wall	Bedding mortar	IV–V	Greyish Brown/no/cohesion loss
	SF09	Funerary enclosure; East stone wall	Bedding mortar	V–VII	Light brown/no/cohesion loss
	SF10	Funerary enclosure; West stone wall	Bedding mortar	V–VII	Light brown/no/cohesion loss
	SF11	Funerary enclosure; North stone wall	Bedding mortar from foundations	V–VII	Light brown/no/cohesion loss
	SF12	Grave LXIII (inside funerary enclosure); brick wall	Bedding mortar	V–VII	Light brown/no/cohesion loss
	SF15	Stone wall	Bedding mortar	II	Brown/no/cohesion loss
	SF16	Grave XLIX (inside funerary enclosure); brick wall	Bedding mortar	IV–V	Light brown/no/cohesion loss
	SF19	Grave LXXXV; brick wall	Bedding mortar	V–VII	Greyish brown/no/cohesion loss
Coating and floor mortars (CFM)	SF01	Grave CCX; brick wall	*Opus signinum*; coating mortar—from the wall	IV–V	Reddish/cohesion loss
	SF02	Grave CCX; brick wall	*Opus signinum*; coating mortar with brick fragments from the top of the cover (may act as impermeabilization render)	IV–V	Pink or reddish brown/paraloid 10%/cohesion loss
	SF04	Grave LXXXV	*Opus signinum*; floor mortar, uppermost layer	V–VII	Reddish/cohesion loss
	SF05	Grave LXXXV	Preparatory layer; floor mortar, lowermost layer	V–VII	Reddish/no/cohesion loss
	SF06	Grave LXXXV; brick wall	*Opus signinum*; coating mortar from the top of the cover (may act as impermeabilization render)	V–VII	Orange or reddish/no/cohesion loss
	SF07	Grave LXXXV; brick wall	*Opus signinum*; coating mortar from the wall	V–VII	Reddish/cohesion loss
	SF08	Grave LVII; stone wall	*Opus signinum*; coating mortar from the wall	V–VII	Reddish brown/no/cohesion loss
Bricks (B)	SF13	Grave LXIII (inside funerary enclosure); brick wall	Brick from cover	IV–V	Orange/no
	SF14	Grave XLIX (inside funerary enclosure); brick wall	Brick from the wall	IV–V	Orange/no
	SF17	Grave CCX; brick wall	Brick (*lydion* type) from the wall	IV–V	Orange/no
	SF18	Grave LXXXV; brick wall	Brick from the wall	V–VII	Orange/no

**Table 2:** Samples major elements chemical composition, in %.

Group		Al_2_O_3_	CaO	Fe_2_O_3_	MgO	K_2_O	MnO	Na_2_O	P_2_O_5_	SiO_2_	SO_3_	TiO_2_	LOI
Masonry mortars (MM)	Min	20.77	0.51	5.37	1.43	5.80	0.06	0.33	0.38	50.07	0.03	1.11	4.78
	Max	23.99	0.96	7.23	1.66	6.73	0.11	0.89	1.82	56.57	0.11	1.19	6.54
	Mean	22.88	0.75	6.16	1.53	6.30	0.08	0.62	0.94	53.80	0.07	1.15	5.31
	SD	1.29	0.19	0.58	0.07	0.31	0.02	0.22	0.54	1.88	0.03	0.03	0.56
Coating and floor mortars (CFM)	Min	18.28	0.35	3.51	0.90	3.82	0.07	0.28	0.36	42.26	0.05	0.65	3.61
	Max	29.78	0.99	7.61	1.45	6.61	0.11	0.75	4.53	63.83	0.32	1.25	7.89
	Mean	25.96	0.54	6.35	1.20	4.94	0.08	0.45	1.86	51.41	0.17	1.09	5.35
	SD	4.52	0.21	1.48	0.21	1.07	0.01	0.22	1.33	7.00	0.11	0.21	1.38
Bricks (B)	Min	28.27	0.12	6.33	0.54	3.70	0.02	0.14	0.31	50.38	0.02	1.10	1.36
	Max	32.83	0.33	8.52	0.82	3.85	0.04	0.20	1.03	56.01	0.12	1.23	6.04
	Mean	30.17	0.23	7.02	0.69	3.80	0.03	0.17	0.68	52.07	0.05	1.18	3.63
	SD	2.01	0.09	1.02	0.11	0.07	0.01	0.03	0.35	2.65	0.05	0.06	2.48

Min: minimum; Max: maximum; Sd: Standard deviation; LOI: Loss of ignition.

**Table 3:** Samples trace elements chemical composition, in mg/kg.

Group		As	Ba	Br	Cl	Cr	Cu	Nb	Nd	Ni	Pb ^1^	Sn	Sr	V	Y	Zn	Zr
Masonry mortars (MM)	Min	5.9	720.0	6.8	120.0	21.9	32.3	15.7	61.1	8.1	46.9	10.4	140.0	56.4	13.0	57.5	370.0
	Max	21.4	900.0	16.9	370.0	89.8	210.0	18.4	80.8	9.8	220.0	21.4	180.0	70.1	18.7	78.9	410.0
	Mean	10.9	820.0	10.5	222.5	38.2	85.7	17.1	69.1	8.8	94.5	12.8	163.8	64.7	15.1	66.6	386.3
	SD	4.3	61.4	3.2	98.8	20.5	58.4	0.9	5.5	0.6	64.8	3.6	14.9	4.0	1.7	5.6	13.2
Coating and floor mortars (CFM)	Min	5.4	400.0	3.9	160.0	28.4	27.9	11.7	24.3	6.3	43.5	8.1	67.6	27.3	10.8	46.0	180.0
	Max	28.1	820.0	62.0	610.0	65.4	160.0	28.8	91.5	16.1	180.0	34.1	150.0	90.0	71.5	110.0	460.0
	Mean	14.9	565.7	22.5	357.1	50.8	67.8	21.3	62.1	11.5	81.6	19.7	85.6	62.1	35.0	72.2	348.6
	SD	7.5	136.3	17.9	166.2	11.0	41.5	6.8	20.0	3.4	46.7	7.7	26.9	18.0	19.7	21.1	90.5
Bricks (B)	Min	11.9	270.0	6.3	40.0	46.5	20.3	27.5	38.6	9.9	42.6	19.8	35.6	51.7	30.1	56.7	370.0
	Max	34.7	340.0	16.7	280.0	54.3	31.4	34.4	56.9	13.6	63.6	35.1	89.8	67.6	45.2	74.7	590.0
	Mean	22.4	307.5	10.8	146.7	50.4	28.6	32.0	48.5	12.5	51.6	28.1	52.6	56.7	39.3	65.3	465.0
	SD	8.2	32.7	4.4	99.8	2.8	4.8	2.6	6.6	1.5	7.9	5.7	21.8	6.4	6.1	7.2	93.4

SD: Standard deviation; Min: minimum; Max: maximum; LOI: Loss of ignition. ^1^ The Pb content of SF08 (17,890 mg/kg) sample, from coating and floor mortars group was not included in the statistical analysis.

## Data Availability

Not applicable.
